# Correlation between triglyceride glucose-body mass index and hypertension risk: evidence from a cross-sectional study with 60,283 adults in eastern China

**DOI:** 10.1186/s12872-024-03934-8

**Published:** 2024-05-23

**Authors:** Yijia Chen, Jinling Du, Nan Zhou, Yingqian Song, Weiwei Wang, Xin Hong

**Affiliations:** 1 Department of Chronic and Noncommunicable Disease Prevention, Nanjing Medical University Affiliated Nanjing Municipal Center for Disease Control and Prevention, Nanjing, 210003 China; 2 Guangzhou Liwan Center for Disease Control and Prevention, Guangzhou, 510176 China; 3 Department of Epidemiology and Biostatistics, School of Public Health, Nanjing Medical University, Nanjing, 211166 China; 4 Nanjing Gulou Center for Disease Control and Prevention, Nanjing, 210015 China

**Keywords:** Hypertension, Triglyceride, Body mass index, TyG-BMI, Triglyceride glucose-body mass index

## Abstract

**Background:**

Insulin resistance (IR) and obesity are established risk factors for hypertension, with triglyceride-glucose (TyG) serving as a recognized surrogate marker for IR. The aim of this study was to investigate the association between TyG-BMI and hypertension in the general population.

**Methods:**

A total of 60,283 adults aged ≥18 years who underwent face-to-face questionnaires, anthropometric measurements, and laboratory examination were included in this study. Multivariable logistic regression models and receiver operating characteristic curve (ROC) were used to determine the association between TyG-BMI and hypertension. The restricted cubic spline model was used for the dose-response analysis.

**Results:**

After fully adjusting for confounding variables, multivariate logistic regression model showed a stable positive association between TyG-BMI and hypertension (OR: 1.61 per SD increase; 95% CI: 1.55–1.67; P-trend < 0.001). The multivariate adjusted OR and 95% CI for the highest TyG-BMI quartile compared with the lowest quartile were 2.52 (95% CI 2.28–2.78). Dose-response analysis using restricted cubic spline confirmed that the association between TyG-BMI index and hypertension was linear. Subgroup analyses showed that stronger associations between TyG-BMI index and hypertension were detected in young and middle-aged individuals (P for interaction < 0.05). ROC analysis showed that TyG-BMI index could better predict the risk of hypertension than other parameters (TyG-BMI cut-off value: 207.105, AUC: 0.719, sensitivity 65.5%, specificity 66.8%), particularly among young and middle-aged people.

**Conclusion:**

The TyG-BMI index was independently associated with hypertension in the study population. Further studies are required to confirm this relationship.

**Supplementary Information:**

The online version contains supplementary material available at 10.1186/s12872-024-03934-8.

## Introduction

Hypertension, a major risk factor for cardiovascular disease (CVD) and premature death, has been recognized as a worldwide public health challenge [1, 2]. A recent national survey found that the crude prevalence of hypertension among Chinese residents aged ≥ 18 years was 27.9% (weighted rate of 23.2%) [3]. The direct economic burden due to hypertension accounted for 6.6% of total health costs in China, imposing a significant burden of disease on the country [4]. Given the large number of people suffering from hypertension and the heavy burden of the disease, it would be meaningful to identify at-risk individuals prone to hypertension at an early stage through a simple but effective diagnostic tool.

Insulin resistance (IR) is a common pathological state in which insulin-dependent cells, such as adipocytes and cardiomyocytes, are impaired in their ability to respond to insulin [5]. Several studies have demonstrated that IR is the key mechanism in many metabolic diseases, such as diabetes [6], obesity [7] and metabolic syndrome [8]. In addition, a meta-analysis involving 11 studies showed that IR is independently associated with hypertension and plays a crucial role in the development of hypertension [9]. Several studies [10, 11] have shown that IR causes elevated blood pressure by causing renal sodium retention, activating the sympathetic nervous system, increasing peripheral and renal vascular resistance, and damaging the vascular endothelium. Therefore, early and accurate recognition of IR is clinically important for the implementation of preventive and management measures for hypertension. The hyperinsulinemic-euglycemic clamp (HEC) technique is currently the gold standard for the diagnosis of insulin resistance [12]. However, this method of assessing IR is costly, complex, time-consuming and ethically restricted.

In recent years, the triglyceride glucose (TyG) index, calculated from the combination of fasting glucose (FPG) and triglycerides (TG), has been widely used as a simple and effective surrogate marker for the early identification of IR [13–16]. Currently, it has been shown that the product of TyG index combined with body mass index (BMI) has a stronger diagnostic value in identifying IR [17, 18]. Previous studies have highlighted the robust predictive capability of TyG-BMI for major chronic diseases with substantial burden, such as diabetes [19], and cardiovascular diseases, primarily including ischemic stroke, heart failure, and hypertension [20–25]. Furthermore, it has shown a significant ability to predict mortality risk [26]. A cross-sectional study [23] involving 2,124 subjects aged 18 years and older in Romania showed an association between TyG-BMI index and hypertension (OR: 2.12, 95% CI: 1.62–2.78). Another study [24] involving 4,352 Chinese people aged ≥ 65 years also showed that TyG-BMI index was significantly associated with hypertension (OR: 3.56, 95% CI: 2.70–4.70). However, the sample sizes of these studies were relatively small and their study subjects were not representative of the eastern Chinese population aged ≥ 18 years.

Therefore, this study aimed to investigate the association and dose-response relationship between TyG-BMI and hypertension among people aged ≥ 18 years in eastern China.

## Methods

### Study population and sampling

Data were obtained from the Chronic Disease and Risk Factor Surveillance in Nanjing, the capital of Jiangsu Province in eastern China. This survey was a population-based cross-sectional study designed to determine the prevalence of chronic diseases and associated risk factors from January 2017 to June 2018. Detailed information on the study design, sampling methods, participant characteristics have been previously published [27]. Briefly, a representative sample of permanent residents aged ≥ 18 years that had lived in the local village/community for at least 6 months was obtained in this study using a multi-stage stratified random cluster sampling method. A total of 62,000 study subjects were recruited and 61,098 subjects agreed to participate in the survey, with a response rate of 98.5%. Eight hundred and fifteen study subjects were excluded due to missing data from face-to-face questionnaires, anthropometric measurements, and blood samples, 60,283 participants were included in the final analyses.

### Data collection and measurement

Data from face-to-face questionnaires, anthropometric measurements, and blood samples were collected by trained medical professionals. The questionnaire included basic demographic characteristics (e.g., age, gender), behavioral risk factors (e.g., smoking and drinking status), personal and family medical history of chronic diseases (e.g., diabetes, hypertension, dyslipidemia). Anthropometric measurements included height, body weight, waist circumference (WC), and blood pressure (BP). All participants were asked to rest for at least 5 min before BP measurements. BP was measured three times in a seated position by a trained staff using an automatic sphygmomanometer (Omron HBP-1300, Japan). Three measurements were taken at 2-min intervals, and the average of the last two measurements was taken as the final blood pressure value. Blood samples were drawn after a fasting overnight of at least 10 h. Fasting plasma glucose (FPG) was assessed with a glucose oxidase method; total cholesterol (TC), triglycerides (TG), high-density lipoprotein cholesterol (HDL-C), and low-density lipoprotein cholesterol (LDL-C) were analyzed enzymatically with commercially available reagents. The method and study design have been described previously [27].

### Definition of TyG-BMI index

TyG-BMI index = TyG index × BMI, where BMI = weight (kg) / height (m)^2^, and the TyG index = Ln [FPG (mg/dL) × TG (mg/dL) /2] [28].

### Diagnosis of hypertension

According to Chinese guidelines on the prevention and treatment of hypertension in adults (2018 Revised Edition) [29], hypertension was defined as self-reported current treatment with antihypertensive medication in the past two weeks, and/or an average systolic blood pressure (SBP) ≥ 140 mmHg and/or an average diastolic blood pressure (DBP) ≥ 90 mmHg.

### Covariates

According to Chinese guidelines on the prevention and treatment of dyslipidemia in adults (2016 Revised Edition) [30], abnormal TC was defined as TC ≥ 6.2mmol/L, abnormal TG was defined as TG ≥ 2.3mmol/L, abnormal LDL-C was defined as LDL-C ≥ 4.1mmol/L, abnormal HDL-C was defined as HDL-C < 1.0mmol/L. Dyslipidemia was defined as self-reported history of dyslipidemia and/or the use of antilipemic medication, and/or having at least one of the above abnormal serum lipids. According to Chinese guidelines on the prevention and treatment of type 2 diabetes in adults (2017) [31], Diabetes was defined as self-reported current treatment with anti-diabetes medication (insulin or oral hypoglycemic agents), and/or FPG ≥ 7.0 mmol/L. Regular exercise was defined as exercising 2 or more days per week [32, 33]. Current smoking was defined as participants who have smoked at least 100 cigarettes in their lifetime and currently smoke cigarettes [34]. Current drinking was defined as consuming at least 1 alcoholic beverage per week in the past month [35]. Overweight and obesity were defined as BMI between 24.0 and 27.9 kg/m^2^ and of ≥ 28.0 kg/m^2^, respectively [36]. Central obesity was defined as a WC ≥ 90 cm in men and ≥ 85 cm in women [37].

### Statistical analysis

Standardized values (means, prevalence) were calculated using the weight coefficients to represent the total Nanjing adult population aged ≥ 18 years. Weight coefficients accommodated the sampling scheme for unequal probabilities of sample selection, as well as the post-stratification weights, which harmonized the standard population of the 2009 Nanjing Sixth National Population Census by two genders and 12 age groups (5-year intervals) [38]. Quantitative data were presented as means±SD, and qualitative data as proportions. Differences in quantitative and qualitative variables were compared by One-way ANOVA and Chi-square test, respectively.

Multivariate logistic regression model was used to analyze the association between TyG-BMI index and hypertension. Before building the multiple regression models, the collinearity between the variables was checked and the variance inflation factor (VIF) was calculated for each variable. Variables with VIF > 5 were considered as collinear variables and could not be included in the multiple regression model [39]. The results showed that height, weight, BMI, and TG were not included in the model (Supplementary Table 1). As a categorical variable classified by quartile, or a continuous variable using the standard deviation transformed, the level of TyG-BMI index was included in the regression model analysis separately. Four models were used, with the crude model being unadjusted. Model 1 adjusted for sex and age; model 2 adjusted for model 1 plus area, education, current smoking, current drinking, regular exercise, family history of hypertension; model 3 adjusted for age, sex, area, education, current smoking, current drinking, regular exercise, family history of hypertension, WC, TG, LDL-C, HDL-C, FPG. In addition, stratified analysis and interaction test were used to explore whether the correlation between TyG-BMI and hypertension differed between subgroups.

Meanwhile, we calculated the area under the receiver-operating characteristics (ROC) curves (AUC) and other parameters to examine the relationship between TyG-BMI and hypertension. Additionally, we used restricted cubic splines model to analyze the dose-response relationship between TyG-BMI and hypertension [40].

To validate the stability of the results, we conducted sensitivity analysis. Firstly, the participants were classified into three groups based on blood pressure values: ideal blood pressure, pre-hypertension, and hypertension. Using those with ideal blood pressure as the reference group, we analyzed the relationship between TyG-BMI index and pre-hypertension as well as hypertension. Additionally, a multiple linear regression model was employed to analyze the relationship between TyG-BMI index with SBP and DBP.

All statistical analyses were conducted by the R software 3.6.3 (https://www.R-project.org, The R Foundation) or SPSS software (version 20; IBM, Armonk, NY, USA). All *P*-values were two-tailed with a significant level of < 0.05.

## Results

### Baseline characteristics

A total of 60,283 participants (29,848 male and 30,435 female) aged 18 years and above were included in our study. The mean age was 46.86 ± 17.30 years (male mean age was 46.67 ± 17.21 years and female mean age was 47.04 ± 17.38 years). The average TyG-BMI level was 204.86 ± 35.28. There were 15,686 hypertension patients with a weighted prevalence rate of 29.8%. (Supplemental Table 2). The weighted prevalence rate of hypertension is higher among males, the elderly, residents in rural areas, individuals with a primary education or below, current smokers, current drinkers, participants with no regular exercise, and individuals who are overweight or obese, as well as those who have comorbidities of diabetes and dyslipidemia. (All *P* < 0.05) (Supplemental Table 3).

Baseline characteristics according to the TyG-BMI levels were shown in Table 1. Age, Height, Weight, BMI, WC, SBP, DBP, FPG, TC, TG, and LDL-C levels increased with increasing levels of TyG-BMI, while the level of HDL-C decreased with increasing levels of TyG-BMI (All *P* < 0.001). Moreover, participants with higher levels of TyG-BMI had a significantly higher prevalence of obesity/overweight, central obesity, diabetes, dyslipidemia and hypertension (All *P* < 0.05). Subjects with higher TyG-BMI levels had a significantly higher proportion of current smoking, current drinking and family history of hypertension (All *P* < 0.05).

**Table 1:** Baseline characteristics according to TyG-BMI levels

Variable	TyG-BMI quartiles				*P*_trend_ Value
	Q1 (92.16-<178.17)	Q2 (178.17-<199.80)	Q3 (199.80-<223.96)	Q4 (223.96-439.88)	
N	15,063	15,079	15,069	15,072	
TyG-BMI index	161.93±12.11	189.27±6.15	211.16±6.86	250.33±24.03	< 0.001
TyG	8.13±0.45	8.43±0.41	8.66±0.43	9.07±0.60	< 0.001
Age(years), mean ± SD	38.52±16.92	45.49±16.91	49.65±16.49	52.70±15.67	< 0.001
Height (cm), mean ± SD	164.97±7.64	165.74±8.01	165.79±8.78	166.29±8.34	< 0.001
Weight (kg), mean ± SD	54.50±6.55	62.01±7.01	67.76±7.75	76.09±10.26	< 0.001
WC (cm), mean±SD	73.71±7.22	79.16±6.97	83.50±7.39	89.66±8.76	< 0.001
BMI (kg/m²), mean ± SD	19.98±1.53	22.50±1.22	24.44±1.33	27.64±2.52	< 0.001
SBP (mmHg), mean ± SD	117.78±22.37	122.14±16.91	126.04±16.18	131.26±18.55	< 0.001
DBP (mmHg), mean ± SD	73.93±13.19	76.32±12.28	78.41±14.29	81.49±15.64	< 0.001
FPG (mmol/L), mean ± SD	4.82±0.84	5.10±1.21	5.36±1.33	5.98±2.11	< 0.001
TC (mmol/L), mean ± SD	4.32±1.01	4.50±1.10	4.65±1.11	4.94±1.21	< 0.001
TG (mmol/L), mean ± SD	0.97±0.45	1.23±0.55	1.49±0.72	2.24±1.76	< 0.001
HDL-C (mmol/L), mean ± SD	1.54±0.51	1.49±0.52	1.46±0.52	1.38±0.53	< 0.001
LDL-C (mmol/L), mean ± SD	2.40±0.78	2.58±0.83	2.70±0.86	2.86±0.91	< 0.001
Male, n (%)	5057 (33.4)	7171 (46.7)	8590 (56.3)	9030 (59.1)	< 0.001
Urban, n (%)	9764 (65.6)	9972 (66.7)	9865 (66.2)	9213 (61.9)	< 0.001
Education, n (%)					< 0.001
Primary school and lower	714 (6.3)	1173 (9.6)	1455 (11.5)	2116 (16.5)	
Junior or Senior high school	5334 (38.5)	6287 (44.2)	7260 (49.8)	8139 (54.4)	
College and higher	9015 (55.2)	7619 (46.2)	6354 (38.7)	4817 (29.1)	
Current smoking, n (%)	1678 (11.4)	2417 (16.3)	3205 (21.2)	4130 (26.9)	< 0.001
Current drinking, n (%)	3367 (21.9)	4066 (26.6)	4827 (31.5)	5392 (35.1)	< 0.001
Family history of hypertension, n (%)	4158 (32.0)	4760 (37.2)	5135 (40.9)	5958 (47.2)	< 0.001
Regular exercise, n (%)	6726 (44.7)	6863 (44.8)	6889 (44.8)	6553 (42.8)	0.067
Overweight/Obesity, n (%)	54 (0.4)	1674 (11.1)	9602 (63.4)	14,547 (96.4)	< 0.001
Central obesity, n (%)	593 (4.1)	1622 (11.2)	3858 (26.4)	8866 (59.3)	< 0.001
Dyslipidemia, n (%)	1766 (12.5)	2943 (20.3)	4369 (29.8)	8015 (53.8)	< 0.001
Diabetes, n (%)	420 (3.4)	877 (6.9)	1441 (10.9)	2925 (21.1)	< 0.001
Hypertension, n (%)	1401 (11.7)	2742 (21.3)	4372 (32.5)	7171 (51.1)	< 0.001

TyG-BMI, triglyceride glucose- body mass index; WC, waist circumference; BMI, body mass index; SBP, systolic blood pressure; DBP, diastolic blood pressure; FPG, fasting plasma glucose; TC, total cholesterol; TG, triglyceride; HDL-C, high density lipoprotein cholesterol; LDL-C, low density lipoprotein cholesterol; SD, standard deviation

### Association between TyG-BMI and hypertension

**Fig. 1:**
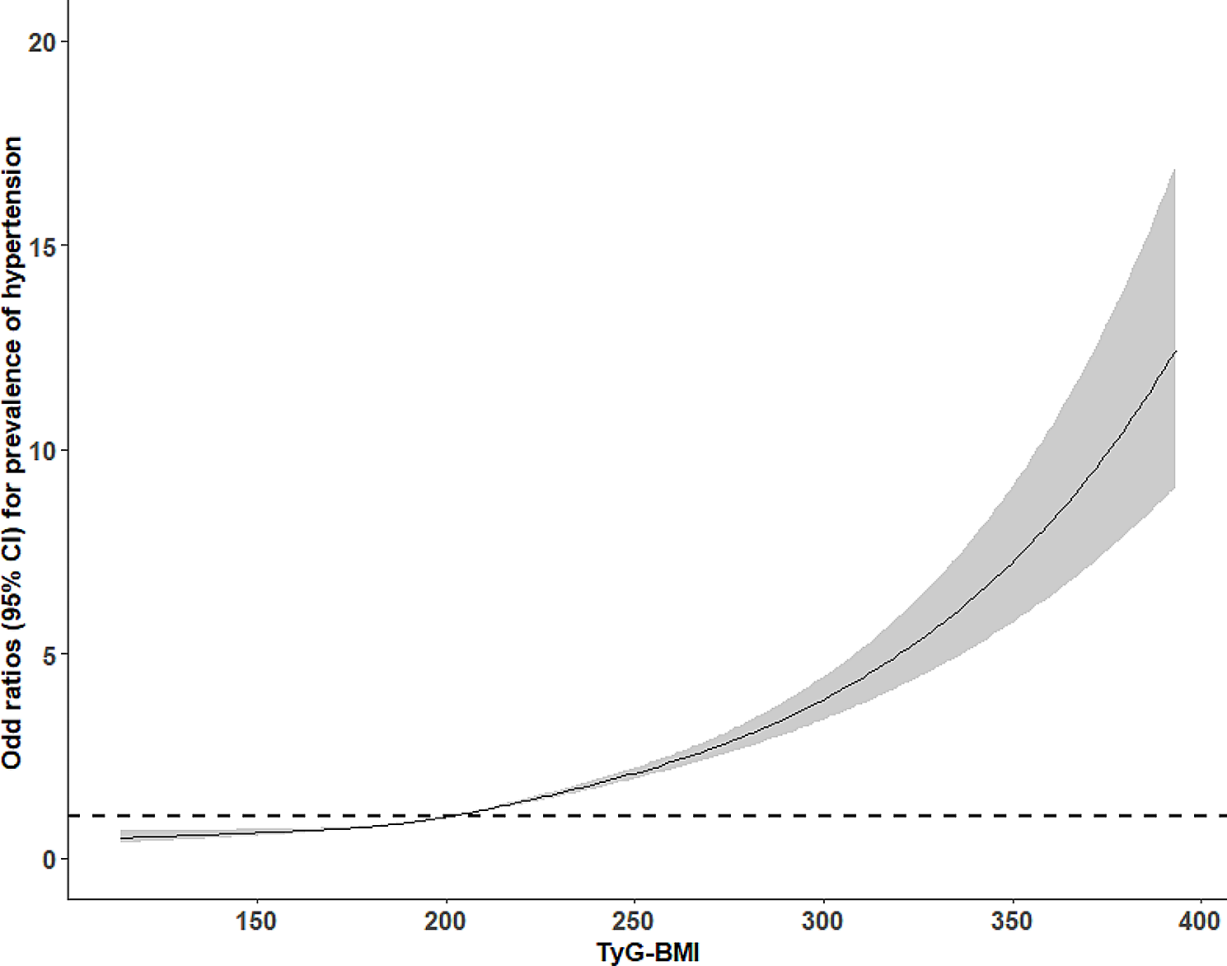
Dose-response relationship between TyG-BMI and hypertension by the restricted cubic spline. Adjusted for age, sex, area, education, current smoking, current drinking, regular exercise, family history of hypertension, WC, TG, LDL-C, HDL-C, FPG

The association between TyG-BMI and hypertension was estimated by a multivariate logistic regression model (Table 2). When the standard deviation-transformed TyG-BMI was analyzed as a continuous variable, per SD increase in TyG-BMI led to higher odds of hypertension in the univariate logistic regression model (OR: 2.24, 95% CI: 2.19–2.28). The association remained statistically significant in all multivariate logistic regression models after adjusting for several covariates including age, sex, area, education, current smoking, current drinking, regular exercise, family history of hypertension, WC, TG, LDL-C, HDL-C, FPG. (Model 1: OR: 1.89, 95% CI: 1.85–1.94; Model 2: OR: 1.82, 95% CI: 1.77–1.87; Model 3: OR:1.61, 95% CI: 1.55–1.67). When the TyG-BMI index was treated as a categorical variable based on quartiles, compared with participants in the lowest quartile, those in the highest quartile of the TyG-BMI index had a higher association with hypertension in all four models (unadjusted model: OR: 8.85, 95% CI: 8.30–9.43; Model 1: OR: 4.67, 95% CI: 4.35–5.02; Model 2: OR:4.16, 95% CI: 3.84–4.51; Model 3: OR: 2.52, 95% CI: 2.28–2.78; all P for trend < 0.001). The dose-response analysis with a restricted cubic spline model showed a linear relationship between the TyG-BMI index and hypertension (P for non-linearity test = 0.006) (Fig. 1). The results of the sensitivity analysis revealed that, when using individuals with ideal blood pressure as the reference group, the multivariate adjusted OR and 95% CI for the highest quartile of TyG-BMI compared to the lowest quartile were 1.88 (95% CI 2.72–2.06) for pre-hypertension and 4.11 (95% CI 3.59–4.70) for hypertension (Supplemental Fig. 1). Additionally, after adjusting for Model 3, a positive association was observed between the TyG-BMI index with SBP and DBP (All P < 0.05) (Supplemental Table 4). In addition, an ROC analysis was conducted to evaluate the value of TyG-BMI in relation to hypertension. Table 3 presented the AUC and optimal thresholds of TyG-BMI and other parameters in association with hypertension. In this dataset, TyG-BMI demonstrated the highest AUC (0.719), indicating a moderate level of performance in relation to hypertension.

**Table 2:** The association between TyG-BMI and hypertension by multivariate logistic regression analysis

TyG-BMI	Odds ratio (95% confidence interval)			
	Crude model	Model 1	Model 2	Model 3
Continuous				
Per SD increase	2.24 (2.19–2.28)	1.89 (1.85–1.94)	1.82 (1.77–1.87)	1.61 (1.55–1.67)
Categorical				
Q1	1	1	1	1
Q2	2.17 (2.02–2.32)	1.45 (1.34–1.57)	1.38 (1.26–1.50)	1.19 (1.09–1.30)
Q3	3.99 (3.73–4.25)	2.18 (2.03–2.35)	2.06 (1.90–2.24)	1.56 (1.43–1.71)
Q4	8.85 (8.30–9.43)	4.67 (4.35–5.02)	4.16 (3.84–4.51)	2.52 (2.28–2.78)
*P* for trend	< 0.001	< 0.001	< 0.001	< 0.001

Model 1: Adjusted for age, sex;

Model 2: Adjusted for age, sex, area, education, current smoking, current drinking, regular exercise, family history of hypertension;

Model 3: Adjusted for age, sex, area, education, current smoking, current drinking, regular exercise, family history of hypertension, WC, TG, LDL-C, HDL-C, FPG.

OR odds ratio, CI confidence interval, SD standard deviation

**Table 3:** Areas under the receiver operating characteristic curves and cut-off point of TyG-BMI and other relevant index

Variable	Cut-off value	Sensitivity	Specificity	AUC	95% confidence interval
TyG-BMI	207.105	0.655	0.668	0.719	0.715–0.724
TyG-WC					
BMI	23.555	0.693	0.593	0.693*	0.688–0.697
FPG	5.295	0.575	0.707	0.690*	0.686–0.695
TyG	8.685	0.558	0.673	0.657*	0.652–0.662
TC	4.795	0.546	0.621	0.610*	0.605–0.616
TG	1.365	0.552	0.609	0.609*	0.604–0.614
TG/HDL-C	1.095	0.465	0.687	0.602*	0.597–0.607
TC/HDL-C	3.435	0.55	0.595	0.596*	0.591–0.610
HDL-C	1.195	0.287	0.755	0.528*	0.523–0.533

AUC, area under the curve; other abbreviations as in Table 1. *P < 0.001, compare with TyG-BMI

### Subgroup analysis

**Fig. 2:**
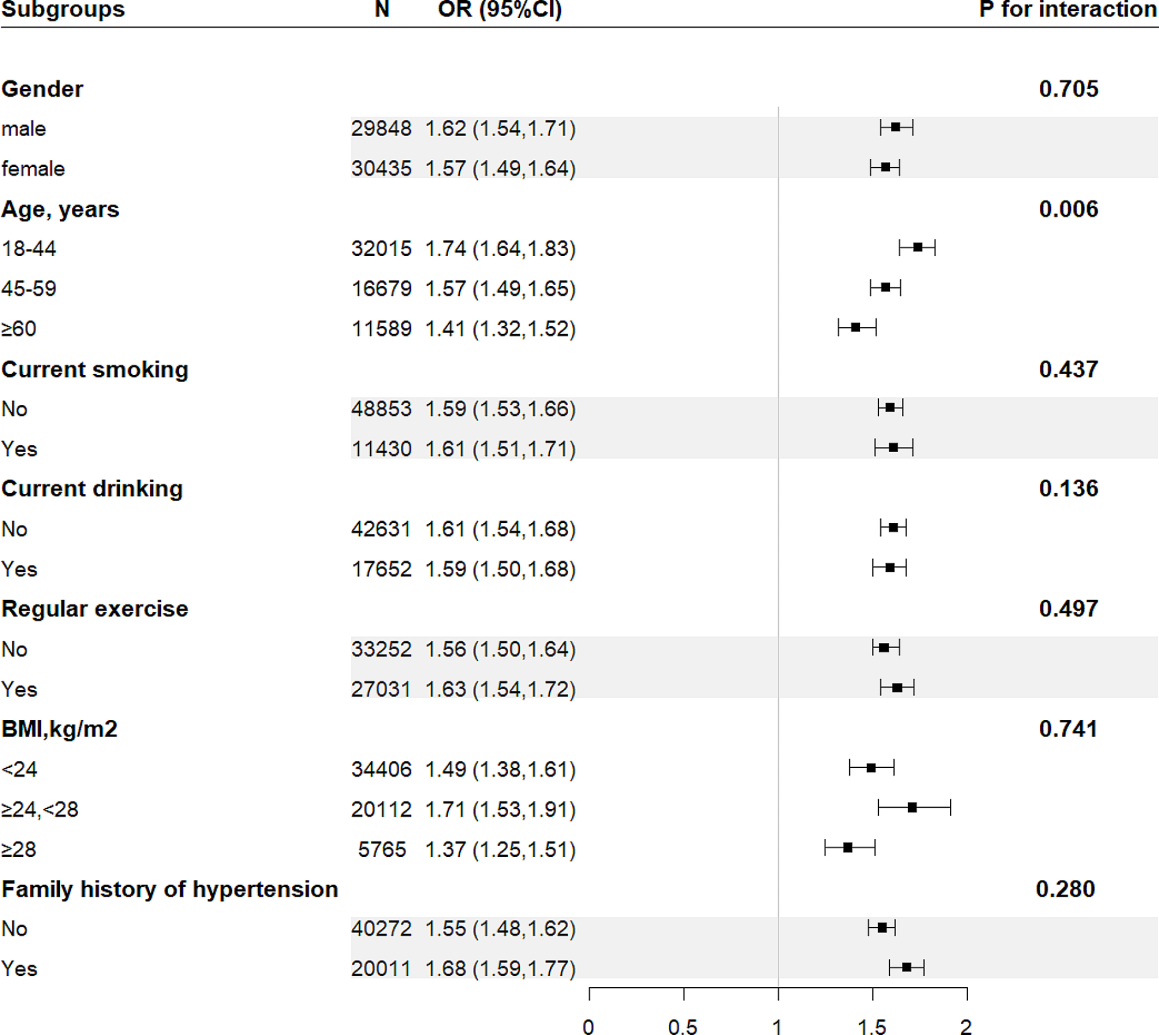
Subgroup analyses of the effect of TyG-BMI index on the prevalence of hypertension. Each subgroup analysis adjusted for age, sex, area, education, current smoking, current drinking, regular exercise, family history of hypertension, WC, TG, LDL-C, HDL-C, FPG, appropriately. Abbreviations as in Table 1

Stratified analysis assessed the the effect of TyG-BMI (per 1 SD increase) on hypertension in different subgroups (Fig. 2). The associations between TyG-BMI and hypertension were positive in the following subgroups: gender (male vs. female; P-interaction = 0.705), current smoking (no vs. yes; P-interaction = 0.437), current drinking (no vs. yes; P-interaction = 0.136), regular exercise (no vs. yes; P-interaction = 0.497), family history of hypertension (no vs. yes; P-interaction = 0.280), and BMI (< 24 kg/m^2^ vs. 24-<28 kg/m^2^ vs. ≥28 kg/m^2^; P-interaction = 0.741).

**Fig. 3:**
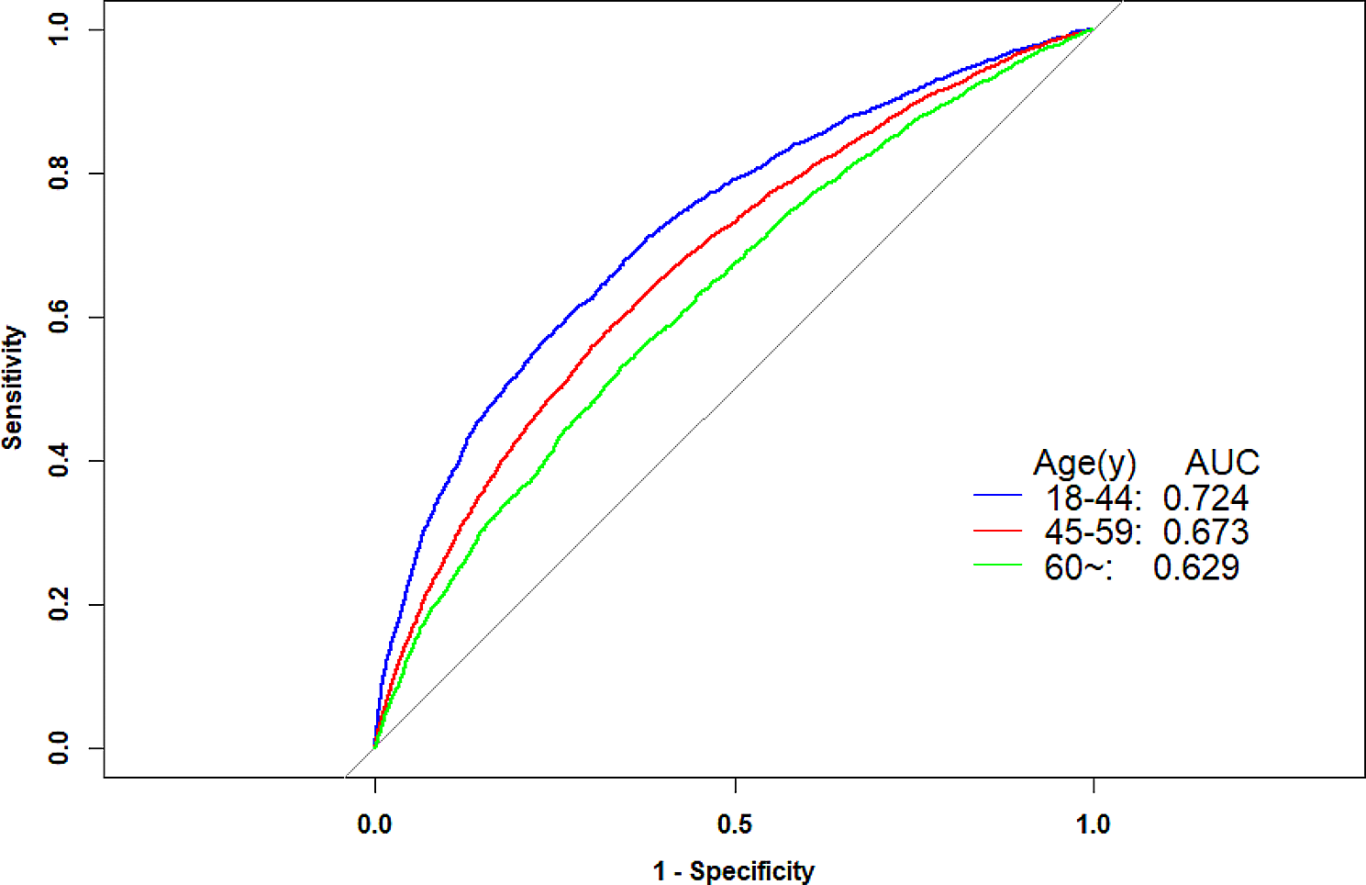
Receiver operating characteristic curve analyses of TyG-BMI by age. All P < 0.05, compare with the group of ≥ 60 years

Moreover, there was a significant interaction between TyG-BMI and age on hypertension (P-interaction < 0.05). In the age stratification, the correlation between TyG-BMI and hypertension was significantly higher in young and middle-aged people than in older people. Considering the significant differences in age stratification in the subgroup analysis. ROC analysis was conducted to further evaluate the association between TyG-BMI and hypertension across different age groups. The results showed that TyG-BMI exhibited a higher AUC value middle-aged and younger individuals with hypertension (Fig. 3, Supplementary Table 5).

## Discussion

In this study, we analyzed the association between TyG-BMI index and hypertension using a large and representative sample. Our results showed that after adjusting for confounders, there was always a significant positive association with hypertension regardless of whether TyG-BMI index was used as a continuous or a categorical variable, showing a nearly linear dose-response relationship. The results of the stratified analysis showed that the direction of the relationships between TyG-BMI and hypertension among different subgroups were consistent with that of the overall study population. The association between TyG-BMI and hypertension was higher in young and middle-aged people than in elderly people. The ROC analysis demonstrated that the relationship between TyG-BMI and hypertension was stronger compared to TyG and BMI.

TyG-BMI index is being extensively studied as a simple and effective new indicator for the diagnosis of insulin resistance. Two cohort studies examined the ability of TyG-BMI to predict prediabetes as well as diabetes, respectively. Their results indicated that the risk of prediabetes and diabetes increased with increasing TyG-BMI. In addition, the ability of TyG-BMI to predict prediabetes and diabetes was higher than that of TyG and BMI [19, 25, 41]. In our study, the association between TyG-BMI index and hypertension was similar to previous studies. Bala C et al. [23] explored the correlation between six surrogate of insulin resistance indexes and hypertension, and their results showed that TyG-BMI was independently associated with hypertension after adjusting for confounders. Li YX et al. studied the association of different insulin resistance surrogates with hypertension and hyperuria, and the results showed that the TyG-BMI index had a stronger correlation with hypertension than TyG index [24]. Moreover, a study [42] involving 105,070 lean adults in China showed that TyG-BMI was significantly associated with pre-hypertension in both men and women. Consistent with previous research findings [16], the present study conducted a further analysis on the positive correlation between TyG-BMI and individuals with pre-hypertension These results suggest that TyG-BMI could serve as an early indicator for identifying pre-hypertension cases. Moreover, ROC analysis in our study demonstrated that the AUC of TyG-BMI surpassed that of TyG and BMI, aligning with prior research outcomes [23, 24]. These findings underscore the predictive potential of TyG-BMI for hypertension.

The results of our subgroup analysis showed significant differences in the association between TyG-BMI and hypertension by age. In the age stratification, the correlation between TyG-BMI and hypertension was significantly higher in young and middle-aged people than in older people. This phenomenon is thought to be the effect of rapid social development and changes in behavioral lifestyles. On the one hand, young and middle-aged people are under tremendous social pressure due to the increasing aging of the population and the decreasing labor force [43–45]; on the other hand, with the accelerated development of urbanization and modernization, young people are increasingly exposed to unhealthy lifestyle habits, such as overeating and lack of physical activity, which lead to the premature appearance of various metabolic problems [46–48]. Previous studies have shown that TyG-BMI index in normal weight individuals was more strongly associated with diabetes [19], prediabetes [41], and NAFLD [49] than overweight and obese individuals. However, this phenomenon did not exist in our study. This can be explained by the inconsistency of the study population and study outcomes. In addition, the diagnosis of obesity in our study was based on BMI. With changes in people’s lifestyle and dietary habits, some subtle changes have taken place in the structure of the human body, especially the significant increase in fat storage [50, 51]. Relying on body mass index alone to distinguish obesity does not reflect this information.

Although the underlying mechanisms of the relationship between TyG-BMI and hypertension are unclear, it may be related to insulin resistance. Some reviews have shown that insulin resistance is the mechanism of the development and progression of hypertension [52, 53]. Although the stimulatory effect of insulin on glucose uptake by adipocytes is severely diminished by insulin resistance, the effect of insulin on salt reabsorption in the proximal tubules of the kidney is preserved. Thus, insulin-resistant individuals promote renal tubular salt absorption due to compensatory hyperinsulinemia, leading to a state of salt overload and hypertension [54]. Moreover, insulin resistance mediates the effect of hyperuricemia on the risk of hypertension, and the combined or synergistic role of hyperuricemia and insulin resistance in the development of cardiovascular disease has been reported documented [55]. Uric acid and IR may affect blood pressure through the following mechanisms: (1) activation of the renin-angiotensin-aldosterone system, (2) direct effects of uric acid and insulin on stimulation of renal sodium reabsorption [56, 57].

The hyperinsulinemic-euglycemic clamp (HEC) technique, the gold standard for the diagnosis of insulin resistance, is not suitable for routine clinical work because of its high cost, time consuming and complicated procedures. Although homeostasis model assessment of IR (HOMA-IR) has a wide range of clinical application, it does have the disadvantage of relatively high cost and low reproducibility of measuring plasma insulin [58]. The relationship between TyG-BMI and HOMA-IR has been proven [17]. Unlike HEC and HOMA-IR, TyG-BMI does not require insulin but only FPG, TG, and BMI, and is a simple and effective indicator for the diagnosis of insulin resistance. This study further revealed that the ROC value of TyG-BMI for hypertension surpassed that of individual markers including TyG or BMI. Consequently, we have reason to believe that TyG-BMI holds promise in identifying high-risk individuals with hypertension. It is recommended that future prospective studies delve deeper into exploring the predictive value of TyG-BMI for hypertension.

This study has several advantages worth mentioning. (1) The study included 60,283 study subjects with a large and representative sample size. (2) After adjusting for confounders, a linear dose-response relationship between TyG-BMI and hypertension was confirmed. Subgroup analysis identified high-risk groups. With these reliable statistical analyses, the conclusions of this study can be considered quite reliable.

Some disadvantages of this research cannot be ignored. (1) This study is a cross-sectional study and can only confirm the association between TyG-BMI and hypertension, but not the causal relationship between TyG-BMI and hypertension. (2) Some study subjects may already know their blood pressure levels and thus have changed their lifestyles at the time of the survey, which may lead to Ney-man bias. This bias may misestimate the association between influencing factors and disease. (3) Given the cross-sectional design of this study, it presents a challenge to determine the temporal relationship between TyG-BMI and the onset of hypertension in the absence of longitudinal data. Consequently, the accurate assessment of TyG-BMI’s predictive value for hypertension is limited. To better understand the temporal association between these variables, longitudinal studies are warranted for further investigation. (4) Due to limitations of the survey, we were unable to compare the TyG-BMI with the hyperinsulinemic-euglycemic clamp (HEC) technique, the gold standard for the diagnosis of insulin resistance.

## Conclusion

This cross-sectional study of a Chinese population showed an independent association between TyG-BMI and hypertension after adjusting for confounders, and this association was more pronounced in young and middle-aged populations. Furthermore, the AUC value of TyG-BMI for hypertension was higher than that of individual markers such as TyG or BMI, suggesting that TyG-BMI may serve as a novel target for the prevention and management of hypertension. Lifestyle modifications, encompassing physical exercise and adopting healthy dietary practices for weight management, play a crucial role in ameliorating IR and mitigating the risk of hypertension.

### Electronic supplementary material

Below is the link to the electronic supplementary material.

Supplementary Material 1

Supplementary Material 2

## Data Availability

The datasets used and/or analyzed during the current study are available from the corresponding author upon reasonable request.

## Abbreviations

TyG-BMI: triglyceride glucose- body mass index
WC: waist circumference
BMI: body mass index
SBP: systolic blood pressure
DBP: diastolic blood pressure
FPG: fasting plasma glucose
TC: total cholesterol
TG: triglyceride
HDL-C: high density lipoprotein cholesterol
LDL-C: low density lipoprotein cholesterol
SD: standard deviation
