# Supplementary material for: Correlation between triglyceride glucose-body mass index and hypertension risk: evidence from a cross-sectional study with 60,283 adults in eastern China
Source: BMC Cardiovasc Disord. 2024 May 23;24:270. doi: 10.1186/s12872-024-03934-8 (PMC11112881; doi:10.1186/s12872-024-03934-8)
Supplement: Supplementary file 1 — Supplementary Material 1 [file 12872_2024_3934_MOESM1_ESM.docx]

**Supplemental Tables**

**Supplemental Table1.** Collinearity diagnostics steps

**Supplemental Table2.**  Baseline characteristics of participants according to hypertension

**Supplemental Table3.** Results of the receiver operating characteristic curve analyses of

TyG-BMI in subgroups of age

**Supplemental Table4.**  The association between TyG-BMI and blood pressure by multiple linear regression model

**Supplemental Table5.** Results of the receiver operating characteristic curve analyses of TyG-BMI for predicting hypertension in subgroups of age

**Supplemental Table1.** Collinearity diagnostics steps

| **Variable** | **VIF** | | | |
| --- | --- | --- | --- | --- |
|  | **Step 1** | **Step 2** | **Step 3** | **Step 4** |
| Age | 1.7 | 1.7 | 1.7 | 1.7 |
| Sex | 2.6 | 2.4 | 2.4 | 2.2 |
| Area | 1.1 | 1.1 | 1.1 | 1.1 |
| Education | 1.5 | 1.5 | 1.5 | 1.5 |
| Current Drinking | 1.3 | 1.3 | 1.3 | 1.2 |
| Current Smoking | 1.4 | 1.4 | 1.4 | 1.4 |
| Regular exercise | 1.0 | 1.0 | 1.0 | 1.0 |
| Family history of hypertension | 1.1 | 1.1 | 1.1 | 1,1 |
| Height | 14.8 | NA | NA | NA |
| Weight | 39.2 | 6.7 | 6.7 | NA |
| BMI | 11.9 | 8.6 | 8.6 | NA |
| WC | 2.1 | 2.1 | 2.1 | 2.1 |
| TC | 1.7 | 1.7 | NA | NA |
| TG | 3.1 | 2.3 | 2.3 | 2.2 |
| HDL-C | 1.1 | 1.1 | 1.1 | 1.0 |
| LDL-C | 1.6 | 1.6 | 1.1 | 1.1 |
| FPG | 1.4 | 1.3 | 1.3 | 1.3 |
| TyG-BMI | 23.3 | 12.5 | 3.2 | 3.2 |

TyG-BMI, triglyceride glucose- body mass index; WC, waist circumference; BMI, body mass index; FPG, fasting plasma glucose; TC, total cholesterol; TG, triglyceride; HDL-C, high density lipoprotein cholesterol; LDL-C, low density lipoprotein cholesterol.

VIF: variance inflation factor;

VIF = 1/(1-R^2^). The variables with VIF>5 will be regarded as collinear variables and cannot be included in the multiple regression model.

**Supplemental Table2.** Baseline characteristics of participants according to hypertension

| **Variable** | **All** | **Non-hypertension** | **Hypertension** | ***P* value** |
| --- | --- | --- | --- | --- |
| N | 60283 | 44597 (70.2) | 15686 (29.8) |  |
| TyG-BMI index | 204.86±35.28 | 197.21±32.09 | 222.85±35.89 | <0.001 |
| TyG | 8.59±0.59 | 8.50±0.55 | 8.81±0.62 | <0.001 |
| Age (years), mean±SD | 46.86±17.30 | 41.22±15.27 | 60.11±14.31 | <0.001 |
| Height (cm), mean±SD | 165.72±8.24 | 166.31±8.05 | 164.32±8.52 | <0.001 |
| Weight (kg), mean±SD | 65.47±11.28 | 64.25±10.97 | 68.34±11.47 | <0.001 |
| WC (cm), mean±SD | 81.61±9.28 | 79.96±8.87 | 85.51±9.07 | <0.001 |
| BMI (kg/m²), mean±SD | 23.71±3.14 | 23.12±2.98 | 25.10±3.08 | <0.001 |
| SBP (mmHg), mean±SD | 124.54±19.21 | 118.79±10.01 | 138.09±27.21 | <0.001 |
| DBP (mmHg), mean±SD | 77.67±14.22 | 74.82±6.86 | 84.37±22.44 | <0.001 |
| FPG (mmol/L), mean±SD | 5.33±1.53 | 5.10±1.33 | 5.90±1.80 | <0.001 |
| TC (mmol/L), mean±SD | 4.61±1.13 | 4.50±1.10 | 4.88±1.17 | <0.001 |
| TG (mmol/L), mean±SD | 1.51±1.14 | 1.40±0.98 | 1.74±1.41 | <0.001 |
| HDL-C (mmol/L), mean±SD | 1.46±0.52 | 1.47±0.52 | 1.44±0.52 | <0.001 |
| LDL-C (mmol/L), mean±SD | 2.64±0.86 | 2.56±0.83 | 2.84±0.91 | <0.001 |
| Male, n (%) | 29848 (49.4) | 21269 (47.3) | 8579 (54.3) | <0.001 |
| Urban, n (%) | 38814 (65.1) | 28964 (65.6) | 9850 (63.9) | <0.001 |
| Education, n (%) |  |  |  | <0.001 |
| Primary school and lower | 5458 (11.1) | 2749 (7.4) | 2709 (20.0) |  |
| Junior or Senior high school | 27020 (47.0) | 17534 (41.9) | 9486 (58.9) |  |
| College and higher | 27805 (41.9) | 24314 (50.7) | 3491 (21.1) |  |
| Current smoker, n (%) | 11430 (19.2) | 7553 (17.3) | 3877 (23.7) | <0.001 |
| Current drinker, n (%) | 17652 (29.0) | 12509 (28.0) | 5143 (31.3) | <0.001 |
| Family history of hypertension, n (%) | 20011 (33.8) | 12539 (32.5) | 7472 (58.0) | <0.001 |
| Regular exercise, n (%) | 27031 (44.2) | 20006 (44.5) | 7025 (43.5) | <0.001 |
| Overweight/Obesity, n (%) | 25877 (44.6) | 15805 (36.5) | 10072 (63.7) | <0.001 |
| Central obesity, (%) | 14939 (26.2) | 8383 (19.5) | 6556 (42.0) | <0.001 |
| Dyslipidemia, n (%) | 17093 (29.8) | 9927 (23.1) | 7166 (45.6) | <0.001 |
| Diabetes, n (%) | 5663 (10.9) | 2055 (5.2) | 3608 (24.4) | <0.001 |
| TyG-BMI, triglyceride glucose- body mass index; WC, waist circumference; BMI, body mass index; SBP, systolic blood pressure; DBP, diastolic blood pressure; FPG, fasting plasma glucose; TC, total cholesterol; TG, triglyceride; HDL-C, high density lipoprotein cholesterol; LDL-C, low density lipoprotein cholesterol; SD, standard deviation. | | | | |

| **Supplemental Table3.** Prevalence of hypertension in different Characteristics (%) | | | | | |
| --- | --- | --- | --- | --- | --- |
| Variable |  | All | Hypertension | χ² | *P* value |
| Sex | male | 29848(49.4) | 8579(32.8) | 227.503 | <0.001 |
|  | female | 30435(50.6) | 7107(26.9) |  |  |
| Age | <35 | 23162(28.7) | 1206(5.2) | 13772.929 | <0.001 |
|  | 35~<45 | 8853(18.5) | 1286(14.3) |  |  |
|  | 45~55 | 10805(19.5) | 3408(31.2) |  |  |
|  | >55 | 17463(33.3) | 9786(58.9) |  |  |
| Area | Urban | 38814(65.1) | 9850(29.3) | 23.42 | <0.001 |
|  | Rural | 21469(34.9) | 5836(30.8) |  |  |
| Education | Primary school and lower | 5458(11.1) | 2709(53.5) | 5358.827 | <0.001 |
|  | Junior or Senior high school | 27020(47.0) | 9486(37.4) |  |  |
|  | College and higher | 27805(41.9) | 3491(15.0) |  |  |
| Current smoker | yes | 11430(19.2) | 3877(36.8) | 457.147 | <0.001 |
|  | no | 48853(80.8) | 11809(28.2) |  |  |
| Current drinker | yes | 17652(29.0) | 5143(32.2) | 125.813 | <0.001 |
|  | no | 42631(71.0) | 10543(28.9) |  |  |
| Family history of hypertension | yes | 20011(33.8) | 7472(40.3) | 1993.599 | <0.001 |
|  | no | 40272(66.2) | 8214(24.5) |  |  |
| Regular exercise | yes | 27031 (44.2) | 7025(29.3) | 0.026 | 0.872 |
|  | no | 33252 (55.2) | 8661(30.2) |  |  |
| BMI level | low/normal | 34406(55.4) | 5614(19.6) | 4523.556 | <0.001 |
|  | overweight | 20112(34.6) | 7107(38.8) |  |  |
|  | obesity | 5765(10.0) | 2965(55.4) |  |  |
| Diabetes | yes | 5663(10.9) | 3608(66.8) | 4612.557 | <0.001 |
|  | no | 54620(89.1) | 12078(25.3) |  |  |
| Dyslipidemia | yes | 17093(29.8) | 7166(45.6) | 3134.436 | <0.001 |
|  | no | 43190(70.2) | 8520(23.1) |  |  |
| BMI, body mass index; | | | | | |

**Supplemental Table4.** The association between TyG-BMI and blood pressure by multiple linear regression model

| Model | SBP | | | DBP | | |
| --- | --- | --- | --- | --- | --- | --- |
|  | B | t | *P* | B | t | *P* |
| Crude model | 0.147 | 70.235 | <0.001 | 0.085 | 53.985 | <0.001 |
| Model 1 | 0.094 | 43.554 | <0.001 | 0.065 | 38.668 | <0.001 |
| Model 2 | 0.089 | 37.582 | <0.001 | 0.059 | 32.626 | <0.001 |
| Model 3 | 0.061 | 20.453 | <0.001 | 0.045 | 20.010 | <0.001 |

Model 1: Adjusted for age, sex;

Model 2: Adjusted for age, sex, area, education, current smoking, current drinking, regular exercise, family history of hypertension；

Model 3: Adjusted for age, sex, area, education, current smoking, current drinking, regular exercise, family history of hypertension, WC, TG, LDL-C, HDL-C, FPG.

**Supplemental Table5.** Results of the receiver operating characteristic curve analyses of TyG-BMI for predicting hypertension in subgroups of age

| TyG-BMI | Cut-off value | Sensitivity | Specificity | AUC | 95%*CI* |
| --- | --- | --- | --- | --- | --- |
| 18-44 | 207.635 | 0.602 | 0.730 | 0.724* | 0.713-0.735 |
| 45-59 | 215.055 | 0.586 | 0.671 | 0.673* | 0.664-0.681 |
| ≥60 | 216.490 | 0.530 | 0.658 | 0.629 | 0.619-0.640 |

**P*<0.05, compare with the group of ≥60 years.
