# Supplementary material for: Correlation between triglyceride glucose-body mass index and hypertension risk: evidence from a cross-sectional study with 60,283 adults in eastern China
Source: BMC Cardiovasc Disord. 2024 May 23;24:270. doi: 10.1186/s12872-024-03934-8 (PMC11112881; doi:10.1186/s12872-024-03934-8)
Supplement: Supplementary file 2 — Supplementary Material 2 [file 12872_2024_3934_MOESM2_ESM.docx]

**
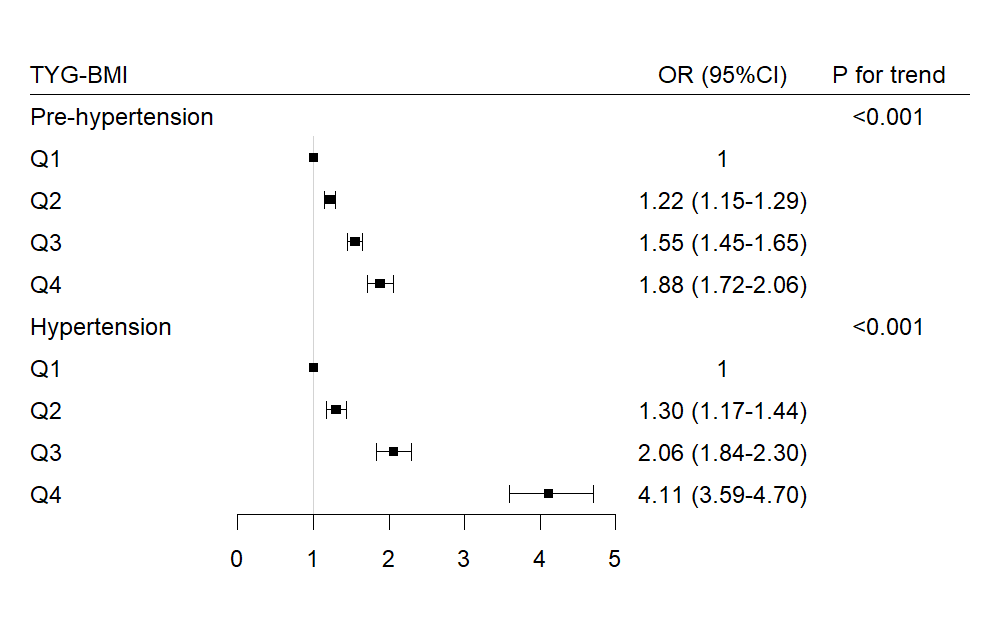
Supplementary Figure1.** The association between TyG-BMI and pre-hypertension/ hypertension. Ideal blood pressure was defined as SBP<120mmHg and DBP<80mmHg, and without history of hypertension; pre-hypertension was defined as 120<SBP<140mmHg or 80<DBP<90mmHg, and without history of hypertension. Model adjusted for age, sex, area, education, current smoking, current drinking, regular exercise, family history of hypertension, WC, TG, LDL-C, HDL-C, FPG.
